# Long-term risk associated with clonal hematopoiesis in patients with severe aortic valve stenosis undergoing TAVR

**DOI:** 10.1007/s00392-022-02135-7

**Published:** 2023-01-21

**Authors:** Silvia Mas-Peiro, Graziella Pergola, Alexander Berkowitsch, Manja Meggendorfer, Michael A. Rieger, Mariuca Vasa-Nicotera, Stefanie Dimmeler, Andreas M. Zeiher

**Affiliations:** 1 Department of Medicine, Cardiology, Goethe University Hospital, Frankfurt am Main, Theodor-Stern-Kai 7, 60590 Frankfurt, Germany; 2 German Centre for Cardiovascular Research (DZHK), Berlin, Germany; 3 Cardio-Pulmonary Institute (CPI), Frankfurt am Main, Germany; 4 Department of Medicine, Hematology/Oncology, Goethe University Hospital, Frankfurt am Main, Germany; 5 MLL Munich Leukemia Laboratory, Munich, Germany; 6 Institute for Cardiovascular Regeneration, Goethe University Frankfurt am Main, Frankfurt am Main, Germany

**Keywords:** Aortic valve disease, TAVR, Clonal hematopoiesis, Inflammation

## Abstract

**Background:**

Mutations in the clonal hematopoiesis of indeterminate potential (CHIP)-driver genes DNMT3A and TET2 have been previously shown to be associated with short-term prognosis in patients undergoing TAVR for aortic valve stenosis. We aimed to extend and characterize these findings on long-term outcome in a large cohort.

**Methods:**

A total of 453 consecutive patients undergoing TAVR were included in an up to 4-year follow-up study. Next-generation sequencing was used to identify DNMT3A- and/or TET2-CHIP-driver mutations. Primary endpoint was all-cause mortality. Since CHIP-driver mutations appear to be closely related to DNA methylation, results were also assessed in patients who never smoked, a factor known to interfere with DNA methylation.

**Results:**

DNMT3A-/TET2-CHIP-driver mutations were present in 32.4% of patients (DNMT3A *n* = 92, TET2 *n* = 71), and were more frequent in women (52.4% vs. 38.9%, *p* = 0.007) and older participants (83.3 vs. 82.2 years, *p* = 0.011), while clinical characteristics or blood-derived parameters did not differ. CHIP-driver mutations were associated with a significantly higher mortality up to 4 years after TAVR in both univariate (*p* = 0.031) and multivariate analyses (HR 1.429, 95%CI 1.014–2.013, *p* = 0.041). The difference was even more pronounced (*p* = 0.011) in never smokers. Compared to TET2 mutation carriers, patients with DNMT3A mutations had significantly less frequently concomitant coronary and peripheral artery disease.

**Conclusion:**

DNMT3A- and TET2-CHIP-driver mutations are associated with long-term mortality in patients with aortic valve stenosis even after a successful TAVR. The association is also present in never smokers, in whom no biasing effect from smoking on DNA methylation is to be expected.

**Graphical Abstract:**

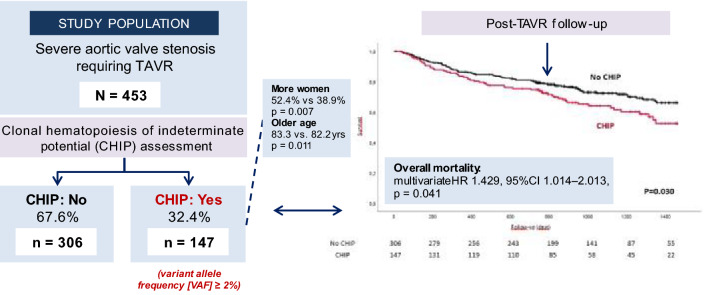

**Supplementary Information:**

The online version contains supplementary material available at 10.1007/s00392-022-02135-7.

## Introduction

Clonal hematopoiesis of indeterminate potential (CHIP), driven by the presence of somatic mutations in hematopoietic cells in patients without other hematological abnormalities, has been shown to have an impact on mortality in patients with cardiovascular diseases [1], including coronary artery disease [2], heart failure [3], and degeneration of aortic valves [4]. More specifically, in a preliminary short-term study by our group, mutations in the most common CHIP-driver genes, DNMT3A and TET2, were shown to be significantly associated with the progression of degenerative aortic valve stenosis (AVS) and to predict a worse clinical short-term outcome even after successful valve replacement by transcatheter aortic valve replacement (TAVR) [4]. Mechanistically, TET2- and DNMT3A-CHIP-driver mutations were experimentally shown to confer increased inflammatory activation of circulating monocytes and macrophages [5]. Recently, using single-cell sequencing analyses, activated inflammatory pathways could also be clinically documented in carriers of DNMT3A- or TET2-CHIP-driver mutations, whose circulating monocytes demonstrate increased expression of IL-1beta, IL-6 receptor, NLRP3 and the macrophage activation receptor [6, 7].

The aim of this study was to extend our prior preliminary short-term results on mortality associated with CHIP in a larger AVS cohort with long-term follow-up. We also aimed at excluding an interfering effect from smoking, a factor known to interfere with DNA methylation, which is also closely related with the CHIP-driver genes, DNMT3A and TET2, on clinical outcomes.

## Methods

### Study cohort

Consecutive patients with severe calcified AVS undergoing TAVR at the University Hospital of the Goethe University Frankfurt between February 2017 and March 2020 were included in the present up to 4-year follow-up study. Patients with hematological disorders (including those with leukemia or lymphoma) were excluded in line with the CHIP definition [8]. Out of 489 patients, 22 patients, who underwent a valve-in-valve procedure for a previously implanted biological valve, were also left out in order to focus solely on patients with native AVS. Preliminary short-term mortality results in 279 of our patients have been previously reported [4]. For mortality analyses, patients with a follow-up shorter than 30 days were excluded (*n* = 14), in order to remove any potential effect of immediate mortality caused by peri-procedural complications (e.g., major/life-threatening bleeding or vascular complication) after TAVR. Taking into account that the most common CHIP-driver genes (DNMT3A and TET2) are related to DNA methylation, and smoking has been also shown to be involved in changes in DNA methylation, separate analyses were performed for never smokers in our population [9].

### Baseline characteristics, laboratory measurements and clinical endpoints

Clinical data, echocardiographic parameters and laboratory findings were collected for all patients. Laboratory measurements included a complete blood count, renal function, and inflammatory parameters, including IL-6 and C-reactive protein. In order to investigate a potential relationship and/or interference of atherosclerotic polyvascular bed disorders with the presence of CHIP-driver mutations on clinical outcomes, baseline atherosclerotic disease was categorized as follows: (a) none; (b) 1 vascular bed involved, i.e., coronary artery disease (CAD); (c) 2 vascular territories involved, i.e., CAD plus either cerebrovascular disease or peripheral arterial occlusive disease (PAOD); and (d) 3 vascular beds affected, i.e., CAD and PAOD and cerebrovascular disease. Prospective follow-up was performed through regular visits in our cardiology outpatient clinic and completed by telephone interviews. The primary clinical endpoint was all-cause mortality.

### Next-generation sequencing (NGS)

Next-generation sequencing (NGS) was performed by MLLSEQ-MLL Dx GmbH, Munich, Germany. Leukocytes/platelets (buffy coat) were buffered in 300 µl RLT buffer (Qiagen, Hilden, Germany), followed by DNA extraction using the Roche MagNA Pure System with the MagNAPure96 DNA and Viral NA LV Kit (Roche LifeScience, Mannheim, Germany). The library preparation for enrichment was performed with 150 ng DNA per sample with the Illumina TruSeq DNA Nano Kit (Illumina, San Diego, CA, USA) using Unique Dual Indices (UDI). Within the protocol, the DNA was fragmented to a length of 150 bp using the Covaris LE220-plus ultrasonicator (Covaris, Woburn, MA, USA). Subsequently, the DNA target regions were enriched using the IDT Hybridization Capture Protocol and a corresponding lockdown gene panel (IDT Integrated DNA Technologies, Coralville, IA, USA). Sequencing of the libraries was performed on Illumina NovaSeq 6000 instruments (Illumina, San Diego, CA, USA) with paired end sequencing mode (2 × 101 cycles) and a target coverage of 4000×. The lockdown panel covered the following genes: DNMT3A and TET2. Illumina’s BaseSpace Enrichment app (v3.1.1) was used to align the raw reads to hg19 reference sequence (Isaac Aligner v03.16.02.20). Subsequently, variants were called using PISCES (v5.1.3.60) somatic variant caller with 2% variant allele frequency (VAF) cutoff and 29 base quality filter and PCR duplicate flagging. In addition, the same data were processed through Illumina’s Dragen Enrichment app (v3.6.3) with 2% VAF, 2% VAF filter threshold and duplicate marking. We combined calls from both result files (VCF) for tertiary analysis. The classification of the variants in mutated, variant of uncertain significance (VUS), or polymorphism was done using the public databases ClinVar, COSMIC, dbSNP, gnomAD, as well as the MLL in-house variant data base.

### Statistical analysis

Mean ± standard deviation or median (IQR) are reported for continuous variables, based on their normal or non-normal distribution. Analysis of variance testing was used for comparison of continuous variables between groups. Frequencies were used for categorical variables and compared by chi-square test or Fisher’s exact test as applicable. Kaplan–Meier curves were plotted, and log-rank test was applied for survival analysis. Multivariate Cox regression analysis was performed in order to adjust for potential confounding factors. Statistical significance was based on a *p* value < 0.05. All analyses were performed with SPSS statistical software package, version 26.0.

## Results

### Detection of mutations and baseline characteristics

DNMT3A- and/or TET2-CHIP-driver mutations with a variant allele frequency (VAF) ≥ 2% were present in 32.4% of patients with AVS (DNMT3A *n* = 92, TET2 *n* = 71). A total of 16 patients showed mutations in both genes. Baseline characteristics, echocardiographic findings and laboratory parameters according to CHIP-driver mutation status are shown in Table 1. Almost half of the population were female patients; median age of the overall population was 82.7 (79.1–85.8) years, with hypertension being the most common comorbidity among patients, followed by atrial fibrillation. The proportion of women was significantly greater in the group of patients carrying CHIP-driver mutations (52.4% vs 38.9%, *p* = 0.007). Patients carrying a DNMT3A- or a TET2-CHIP-driver mutation were significantly older than those without any mutation (83.3 vs. 82.2 years, *p* = 0.011). A total of 86 patients (19.0%) had a history of previous cancer therapy (most commonly: prostate and breast cancer). Out of all patients with a history of cancer, 26 patients had a CHIP-driver mutation whereas 60 patients had no CHIP mutation. No statistically significant difference was found in cancer history between patients with versus without CHIP-driver mutations (Table 1). All other assessed comorbidities did not differ between CHIP-carriers and non-carriers. Interestingly, no differences in inflammatory serum markers were found between both groups, and all other laboratory parameters were also similar in both groups.

**Table 1:** Baseline characteristics, echocardiographic findings and laboratory parameters in patients with and without DNMT3A/TET2-CHIP-driver mutations (*N* = 453)

	Total cohort (*n* = 453)	DNMT3A/TET2 (*n* = 147)	No-DNMT3A/TET2 (*n* = 306)	*p* Value
*Clinical/echocardiographic characteristics*				
Age (years) (*n* = 453)	82.7 (79.1–85.8)	83.3 (80.0–86.5) (*n* = 147)	82.2 (78.8–85.3) (*n* = 306)	**0.011**
Sex (female) (%) (*n* = 453)	43.3% (*n* = 196)	52.4% (*n* = 77)	38.9% (*n* = 119)	**0.007**
BMI (kg/m^2^) (*n* = 453)	26.5 (23.9–29.8)	25.8 (23.2–29.7) (*n* = 147)	26.7 (24.4–30.0) (*n* = 306)	0.104
Hypertension (%) (*n* = 453)	82.1% (*n* = 372)	81.6% (*n* = 120)	82.4% (*n* = 252)	0.851
Diabetes (%) (*n* = 453)	31.3% (*n* = 142)	33.3% (*n* = 49)	30.4% (*n* = 93)	0.528
Previous MI (%) (*n* = 453)	18.5% (*n* = 84)	18.4% (*n* = 27)	18.6% (*n* = 57)	0.947
Previous PCI (%) (*n* = 453)	40.8% (*n* = 185)	37.4% (*n* = 55)	42.5% (*n* = 130)	0.304
Previous stroke (%) (*n* = 453)	13.0% (*n* = 59)	17.0% (*n* = 25)	11.1% (*n* = 34)	0.081
Carotid artery disease (%) (*n* = 453)	17.2% (*n* = 78)	15.6% (*n* = 23)	18.0% (*n* = 55)	0.539
Peripheral artery disease (%) (*n* = 453)	13.0% (*n* = 59)	10.2% (*n* = 15)	14.4% (*n* = 44)	0.216
COPD (%) (*n* = 453)	20.3% (*n* = 92)	19.0% (*n* = 28)	20.9% (*n* = 64)	0.644
Atrial fibrillation (%) (*n* = 453)	46.4% (*n* = 210)	49.7% (*n* = 73)	44.8% (*n* = 137)	0.329
LVEF (%) (*n* = 453)	60 (45–60)	60 (45–60) (*n* = 147)	60 (50–60) (*n* = 306)	0.607
History of cancer (*n* = 453)	19.0% (*n* = 86)	17.7% (*n* = 26)	19.6% (*n* = 60)	0.625
NYHA (*n* = 453)				0.822
I	1.5 (*n* = 7)	1.4 (*n* = 2)	1.6 (*n* = 5)	
II	19.6 (*n* = 89)	19.7 (*n* = 29)	19.6 (*n* = 60)	
III	69.1 (*n* = 313)	67.3 (*n* = 99)	69.9 (*n* = 214)	
IV	9.7 (*n* = 44)	11.6 (*n* = 17)	8.8 (*n* = 27)	
*Laboratory parameters*				
C-reactive protein (mg/dl) (*n* = 446)	0.32 (0.13–0.86)	0.36 (0.12–0.94) (*n* = 146)	0.31 (0.13–0.82) (*n* = 300)	0.811
Leukocytes (/nl) (*n* = 452)	7.0 (5.8–8.2)	6.8 (5.9–8.3) (*n* = 147)	7.0 (5.9–8.2) (*n* = 305)	0.991
Interleukin 6 (pg/ml) (*n* = 423)	5.8 (3.6–10.9)	5.7 (3.7–11.1) (*n* = 139)	5.8 (3.5–10.9) (*n* = 284)	0.885
Hemoglobin (g/dl) (*n* = 453)	12.2 (10.9–13.4)	12.0 (10.6–13.5) (*n* = 147)	12.3 (11.0–13.4) (*n* = 306)	0.510
Hematocrit (%) (*n* = 451)	35.9 ± 5.1	35.7 ± 5.2 (*n* = 147)	36.0 ± 5.1 (*n* = 304)	0.641
Platelets (/nl) (*n* = 452)	220.5 (179–262)	218 (175.5–251.5) (*n* = 147)	222 (181–269) (*n* = 305)	0.310
Creatinine (mg/dl) (*n* = 453)	1.11 (0.88–1.48)	1.13 (0.88–1.53) (*n* = 147)	1.11 (0.88–1.42) (*n* = 306)	0.746
CK (U/l) (*n* = 448)	72 (51.5–104)	79 (47–105) (*n* = 147)	71 (52–102) (*n* = 301)	0.792

Values are shown in mean ± SD, median (IQR), or %

*BMI* body mass index; *CK* creatinine kinase; *COPD* chronic obstructive pulmonary disease; *LVEF* left ventricular ejection fraction; *MI* myocardial infarction; *NYHA* New York Heart Association; *PCI* percutaneous coronary intervention

Echocardiographic parameters quantifying aortic valve stenosis were assessed. Overall *p* mean value [median (IQR)] was 41 (32–51) mmHg. No statistical differences were found in patients with CHIP-driver mutations versus those with no CHIP mutations [40.5 (32–52)] mmHg versus [41 (32–50) mmHg, *p* = 0.826)]. Results were also similar for aortic valve area (AVA) as measured by continuity equation: overall AVA [median (IQR)] was 0.8 (0.6–0.9) cm^2^ with an identical value for both study groups. Planimetric AVA led to similar conclusions. LVEF did not show differences between groups (Table 1). Thus, we found no indication of a more advanced AVS in either group.

Procedural results and complications are shown in Table 2. Results were very similar in patients with and without CHIP mutations. Overall ICU stay [median (IQR)] was 3 (2–4) days. No statistical differences were found in patients with CHIP-driver mutations versus those with no CHIP mutations (identical value for both study groups). Overall hospital stay [median (IQR)] was 7 (6–11) days. No statistical differences were found in patients with CHIP-driver mutations versus those with no CHIP mutations [7 (6–11) days vs. 8 (6–12) days, *p* = 0.788].

**Table 2:** In-hospital complications according to VARC criteria in patients with and without DNMT3A/TET2-CHIP-driver mutations (*N* = 453)

	Total cohort (*n* = 453)	DNMT3A/TET2 (*n* = 147)	No-DNMT3A/TET2 (*n* = 306)	*p* Value
Major stroke (%) (*n* = 453)	2.0% (*n* = 9)	2.7% (*n* = 4)	1.6% (*n* = 5)	0.480
Major vascular complication (%) (*n* = 453)	5.1% (*n* = 23)	6.8% (*n* = 10)	4.2% (*n* = 13)	0.246
Major bleeding (%) (*n* = 453)	3.8% (*n* = 17)	5.4% (*n* = 8)	2.9% (*n* = 9)	0.190
Cardiac tamponade (%) (*n* = 453)	1.1% (*n* = 5)	0.7% (*n* = 1)	1.3% (*n* = 4)	1.0
Pacemaker implantation (%) (*n* = 453)	15.9% (*n* = 72)	18.4% (*n* = 27)	14.7% (*n* = 45)	0.318
More-than-mild aortic regurgitation (%) (*n* = 434)	8.5% (*n* = 37)	7.2% (*n* = 10)	9.2% (*n* = 27)	0.495

In the never-smoking subgroup of patients, the same differences in sex proportion and age distribution as in the overall population were observed between CHIP-carriers and non-carriers, and laboratory parameters and comorbidities showed a similar uniform distribution (see Table 3). In the subgroup of never smokers with a DNMT3A mutation, the proportion of women was particularly high (63.0% vs. 39.3% in patients with no CHIP-driver mutations, *p* = 0.001), and a lower frequency of prior PCI or peripheral arterial vascular disease was observed (27.2% vs. 43.7%, *p* = 0.029 and 3.7% vs. 13.7%, *p* = 0.035, respectively) (Supplementary Table 1). Such findings were not observed in never smokers carrying TET2 mutations.

**Table 3:** Baseline characteristics, echocardiographic findings and laboratory parameters in never smokers with and without DNMT3A/TET2-CHIP-driver mutations (*N* = 389)

	DNMT3A/TET2 (*n* = 127)	No-DNMT3A/TET2 (*n* = 262)	*p* Value
*Clinical/echocardiographic characteristics*			
Age (years)	83.6 (57–95.8)	82.6 (59.5–96)	**0.045**
Sex (female) (%)	55.9%	39.3%	**0.002**
BMI (kg/m^2^)	25.9 (16.4–46.8)	26.5 (15.0–44.9)	0.507
Hypertension (%)	84.3%	82.1%	0.592
Diabetes (%)	29.9%	31.7%	0.725
Previous MI (%)	15.7%	18.3%	0.531
Previous PCI (%)	33.9%	43.5%	0.064
Previous stroke (%)	17.3%	11.8%	0.139
Carotid artery disease (%)	13.4%	18.7%	0.190
Peripheral artery disease (%)	9.4%	13.7%	0.227
COPD (%)	16.5%	18.3%	0.654
Atrial fibrillation (%)	52.0%	44.3%	0.154
LVEF (%)	60 (20–80)	60 (15–75)	0.685
*Laboratory parameters*			
C-reactive protein (mg/dl)	0.37 (0.01–17.6)	0.32 (0.01–11.9)	0.625
Leukocytes (/nl)	6.81 (3.34–14.3)	6.94 (3.0–23.4)	0.876
Interleukin 6 (pg/ml)	5.9 (1.5–99.2)	6.1 (1.5–157.7)	0.884
Hemoglobin (g/dl)	12.0 (7.2–16.9)	12.15 (7.7–16.0)	0.799
Creatinine (mg/dl)	1.15 (0.45–6.27)	1.1 (0.51–7.11)	0.587

Values are shown in median (IQR) or %

*BMI* body mass index; *CK* creatinine kinase; *COPD* chronic obstructive pulmonary disease; *LVEF* left ventricular ejection fraction; *MI* myocardial infarction; *PCI* percutaneous coronary intervention

### Prognostic significance of DNMT3A- and TET2-CHIP-driver mutations after TAVR

**Fig. 1:**
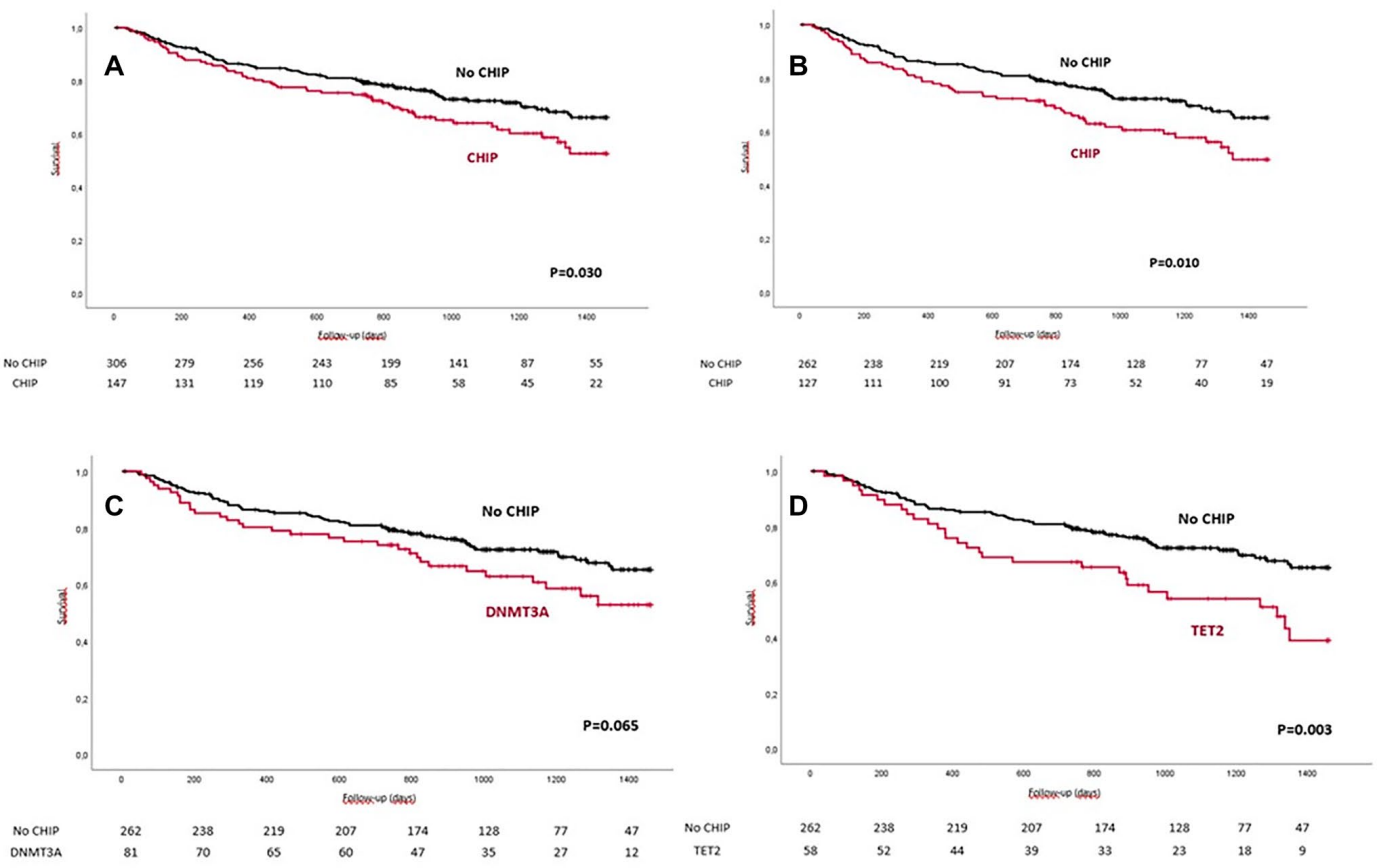
**A**) Overall survival in patients with DNMT3A- or TET2-CHIP-driver mutations with a variant allele frequency >2% vs. patients with no DNMT3A or TET2 mutations. **B**) Overall survival in patients with DNMT3A- or TET2-CHIP-driver mutations with a variant allele frequency >2% vs. patients with no DNMT3A or TET2 mutations in never smokers. **C**) Overall survival in patients with DNMT3A-CHIP-driver mutations with a variant allele frequency >2% vs. patients with no CHIP mutations in never smokers. **D**) Overall survival in patients with TET2-CHIP-driver mutations with a variant allele frequency >2% vs. patients with no CHIP mutations in never smokers. ^*^In order to exclude immediate mortality which is likely to be caused by peri-procedural complications, only patients with a longer than 30-day follow-up were included in the analysis

Overall, patients with a DNMT3A- or TET2-CHIP-driver mutation demonstrated a significantly higher all-cause mortality up to 4 years after TAVR, as shown in the Kaplan–Meier curve (log-rank test, *p* = 0.030) (Fig. 1A). By multivariable Cox regression analysis, the independent association with mortality did persist after taking into account the potential effect of sex, age, LVEF, and EuroSCORE II (HR 1.429, 95%CI 1.014–2.013, *p* = 0.041) (Table 4). This difference was even more pronounced in the never-smoking subgroup (log-rank test = 0.010, see Fig. 1B). When analyzing both mutations separately in never smokers, the higher mortality associated with the presence of CHIP was observed for each mutation with a consistently increasing effect over time (Fig. 1C, D). Significance was achieved in patients carrying a TET2-CHIP-driver mutation (*p* = 0.003), but not in patients with a DNMT3A-CHIP-driver mutation probably due to the relatively small sample size. Interestingly, Kaplan–Meier curves started to diverge at about 1 year in the TET2 group, whereas patients carrying a DNMT3A-CHIP-driver mutation showed curves already diverging at 3 months (Fig. 1C, D).

**Table 4:** Multivariate Cox regression analysis

	*p* Value	HR	95.0%CI for HR	
			Lower	Higher
CHIP	0.041	1.429	1.014	2.013
Age	0.252	1.019	0.987	1.052
Sex	0.508	0.888	0.625	1.262
EuroSCORE II	< 0.001	1.033	1.015	1.050
LVEF	0.010	0.982	0.968	0.996

In line with previous studies reporting a prominent role for TET2-CHIP-driver mutations in atherosclerotic vascular diseases [2], in the never-smoking cohort, atherosclerotic polyvascular bed disease (defined as clinical disorders involving ≥ 2 vascular territories) was present in 34.5% (20/58) of patients carrying TET2 mutations compared with 12.3% (10/81) of patients carrying DNMT3A mutations (*p* = 0.002). However, after adjusting for the presence of each vascular bed, multivariate analysis revealed that carrying a TET2-CHIP-driver mutation remained independently associated with increased mortality (HR 1.943, 95%CI 1.263–2.989, *p* = 0.003).

## Discussion

The results of the present study confirm our preliminary short-term findings and demonstrate that, also in the long term, carrying a DNMT3A- and/or TET2-CHIP-driver mutation is associated with increased mortality risk in patients with severe AVS even after a successful replacement of the calcified stenotic valve by TAVR. Importantly, the increased mortality risk appears to be independent of concomitant atherosclerotic vascular disease and more pronounced in patients who never smoked. Thus, carrying a DNMT3A- or TET2-CHIP-driver mutation appears to be an independent risk factor for worse clinical outcome in patients with severe aortic stenosis even after removal of the stenotic valve by TAVR.

Beyond our previous results that showed CHIP-driver mutations to predict short-term mortality in severe AVS after TAVR, our present findings provide several novel conclusions that are important for the research in the field of cardiovascular implications of CHIP mutations: (1) Until now, only preliminary results in a limited number of patients were available. Confirmation in a large population was clearly needed before any clinical implication may be derived from them. Now, in a large sample, this appears to be confirmed, and the investigation of potentially related factors (such as smoking and atherosclerotic disease or specific mutations) becomes possible. (2) The analysis of CHIP impact on never-smoking patients is particularly important because smoking interferes with DNA methylation, in which CHIP genes are involved. Our data suggest that CHIP mutation effects in AVS are independent from smoking history. (3) CHIP impact does not appear to be explained either by the presence of atherosclerotic polyvascular bed disease. (4) The very high proportion of CHIP mutations we found in severe AVS patients is unparalleled in other populations and will require further investigation. (5) Besides confirming short-term results, Kaplan–Meier curves show a consistently increasing divergence also in the long term, and curves do not tend to become parallel (Fig. 1A). This is important for a mechanistic interpretation in order to differentiate an immediate direct effect of CHIP related to the intervention (and the resulting changes in inflammation) from a background effect on cardiovascular events. (6) The relative significance of the two main mutations and the prominent role of TET2 mutations have been clarified in an AVS population.

Studies in general populations have reported a higher proportion of men in patients carrying CHIP-driver mutations [2, 10]. Although we cannot provide a definite explanation for our findings on sex proportion, our population consisted of patients with severe AVS, which implies they were all very old; this excludes, in fact, pre-/peri-menopausal women. Taking into account the involvement of CHIP in cardiovascular effects of menopause, with CHIP being an independent risk factor for coronary events in postmenopausal women [11, 12], we can speculate on this characteristic of our population being a potential underlying reason. However, we cannot exclude other reasons related to AVS or inflammation processes associated with AVS.

Exposure to cancer therapies has been shown to increase CHIP prevalence [13]. Despite patients with hematological malignancies having been excluded, due to the old age of the vast majority of AVS patients, history of previous cancer therapy was very common in our population. However, no significant effect of cancer history on CHIP mutations could be elicited with our sample size.

Several studies have shown an association between CHIP status and mortality in cardiovascular diseases, specifically in patients with atherosclerosis [2], heart failure of both ischemic and non-ischemic cause [3, 14–16], stroke [17] and, as now demonstrated in the present study, severe aortic stenosis even after removal of the stenotic valve by TAVR.

The increased mortality associated with carrying DNMT3A- and/or TET2-CHIP-driver mutations is independent of traditional risk factors for cardiovascular disease [18]. Mechanistically, experimental studies using CRISPR gene editing revealed that both DNMT3A and TET2-CHIP are associated with aberrant inflammation leading to acceleration of diffuse myocardial fibrosis in pressure-overload or angiotensin II-mediated murine models [7, 19].

As in previous studies, traditional inflammatory biomarkers, CRP and IL-6, were not significantly elevated in the presence of CHIP-driver mutations [4, 20], suggesting that these broad inflammatory serum markers are not sensitive enough to detect subtle differences especially in the elderly patient cohort studied in the present analysis. However, mechanistically, we previously demonstrated a pro-inflammatory signature of circulating monocytes using single-cell transcriptomic profiling of peripheral mononuclear cells from patients undergoing TAVR carrying either DNMT3A or TET2-CHIP-driver mutations, with a higher expression of interleukin 6 receptor, cellular receptor CD163, and the *NLRP3* inflammasome complex [6]. Moreover, our previous study demonstrated that TAVR patients harboring DNMT3A CHIP-driver mutations exhibited a significantly increased ratio of circulating Th17/Treg cells indicating a pro-inflammatory T cell polarization [4]. Pro-inflammatory T cell polarization was not only shown to be associated with increased mortality after TAVR [21], but also correlated with cardiac MRI biosignatures of diffuse interstitial remodeling and fibrosis in chronic coronary artery disease [22]. Diffuse myocardial fibrosis as detected by cardiac MRI is a major determinant of increased all-cause mortality and increased cardiac death after aortic valve replacement [23]. Thus, it is tempting to speculate that an activated pro-inflammatory state in carriers of DNMT3A- and/or TET2-CHIP-driver mutations may have contributed to increased myocardial fibrosis in patients with severe AVS, which confers an increased risk for mortality even after successful removal of the obstructive valve by TAVR.

Strikingly, almost one third of our patients were carriers of a DNMT3A- and/or TET2-CHIP-driver mutation with a variant allele frequency > 2%. Although the prevalence of CHIP-driver mutations is well known to increase with age [1, 24, 25] and the mean age of our patients cohort was almost 83 years, the proportion of patients with severe AVS carrying DNMT3A- and/or TET2-CHIP-driver mutations is considerably higher compared to similar age groups in the normal population [1, 26]. These data suggest that the prevalence of CHIP-driver mutations is significantly increased in patients with severe AVS. Indeed, a recent study has reported that the presence of a CHIP mutation is associated with a significantly increased risk of incident severe AVS with a specific role for DNMT3A-CHIP-driver mutations, which conferred a 2.4-fold increased risk to develop severe AVS in a patient cohort of almost 7000 patients over 40 years of age [27]. Importantly, as smoking is not only well known to associate with CHIP [25, 26, 28, 29], but may itself interfere with DNA methylation [30, 31], we repeated our analyses in patients who never smoked. While the prevalence of DNMT3A- and/or TET2-CHIP-driver mutations remained similar with 32.7%, the association of CHIP with mortality was even stronger in patients with severe aortic stenosis undergoing TAVR, who never smoked. Thus, taken together, both the high prevalence of DNMT3A- and/or TET2-CHIP-driver mutations in patients with severe AVS as well as the increased mortality after successful removal of the stenotic aortic valve appears to be independent of a history of smoking. However, we cannot exclude that the significant mortality observed in the present rather elderly study population may in part also be related to increased epigenetic aging, which was recently shown to profoundly amplify worse clinical outcome in carriers of CHIP mutations [26].

## Limitations

The main limitation of this study is that the exact causes of death could not be retrieved for the entire study population. Furthermore, this is a single-center study, and future studies should be performed at a multicenter level in order to be able to extrapolate results to other populations. On the other hand, the comprehensive assessment of variant allele frequency and the prospective long-term follow-up constitute major strengths of our study. Finally, the high prevalence of CHIP-driver mutations in this elderly population enriched for CHIP does provide for sufficient number of affected patients and events for meaningful statistical analysis.

## Conclusion

In conclusion, carrying a DNMT3A- and/or TET2-CHIP-driver mutation is an independent prognostic risk factor for increased long-term mortality in patients with severe aortic stenosis even after successful removal of the stenotic valve by TAVR. This finding further underscores the need for preventive cardiovascular care for patients with CHIP.

## Supplementary Information

Below is the link to the electronic supplementary material.

Supplementary file1 (DOCX 24 KB)

## Data Availability

Data available on request from the authors.
