# Supplementary material for: Long-term risk associated with clonal hematopoiesis in patients with severe aortic valve stenosis undergoing TAVR
Source: Clin Res Cardiol. 2023 Jan 21;112(5):585–93. doi: 10.1007/s00392-022-02135-7 (PMC10160205; doi:10.1007/s00392-022-02135-7)
Supplement: Supplementary file 1 — Supplementary file1 (DOCX 24 KB) [file 392_2022_2135_MOESM1_ESM.docx]

**Supplementary Table 1**. Baseline characteristics, echocardiographic findings and laboratory parameters in never smokers with DNMT3A-, TET2-, and NO-CHIP-driver mutations (n=401)

|  | **NO-CHIP (n=262)** | | **DNMT3A**  **(n=81)** | | **TET2**  **(n=58)** | | **p-value** | |
| --- | --- | --- | --- | --- | --- | --- | --- | --- |
| ***Clinical/echocardiographic characteristics*** | | | | | | | | |
| **Age (years)** | | 82.6 (59.5-96) | | 83.6 (57.0-95.8) | | 83.6 (73.0-94.3) | | 0.058 |
| **Sex (female) (%)** | | 39.3% | | 63.0% | | 46.6% | | **0.001** |
| **BMI (kg/m^2^)** | | 26.5 (15.0-44.9) | | 25.9 (16.4-46.9) | | 26.9 (18.4-43.0) | | 0.792 |
| **Hypertension (%)** | | 82.1% | | 82.7% | | 87.9% | | 0.557 |
| **Diabetes (%)** | | 31.7% | | 32.1% | | 31.0% | | 0.991 |
| **Previous MI (%)** | | 18.3% | | 16.0% | | 17.2% | | 0.892 |
| **Previous PCI (%)** | | 43.7% | | 27.2% | | 41.4% | | **0.029** |
| **Previous stroke (%)** | | 11.8% | | 16.0% | | 20.7% | | 0.177 |
| **Carotid artery disease (%)** | | 18.7% | | 11.1% | | 17.2% | | 0.281 |
| **Peripheral artery disease (%)** | | 13.7% | | 3.7% | | 15.5% | | **0.035** |
| **COPD (%)** | | 18.4% | | 14.8% | | 19.0% | | 0.737 |
| **Atrial fibrillation (%)** | | 44.3% | | 54.3% | | 48.3% | | 0.278 |
| **LVEF (%)** | | 60 (15-75) | | 60 (20-74) | | 55 (20-80) | | 0.841 |
| ***Laboratory parameters*** | | | | | | | | |
| **C-reactive protein (mg/dl) (n=394)** | 0.32 (0.01-11.9) | | 0.35 (0.01-17.6) | | 0.38 (0.02-5.29) | | | 0.889 |
| **Leukocytes (/nl) (n=400)** | 6.94 (3.0-23.4) | | 7.0 (3.7-14.3) | | 6.5 (3.3-11.5) | | | 0.175 |
| **Interleukin 6 (pg/ml) (n=376)** | 6.1 (1.5-157.7) | | 5.3 (1.5-99.2) | | 5.9 (1.5-45.2) | | | 0.911 |
| **Hemoglobin (g/dl) (n=401)** | 12.15 (7.7-16.0) | | 12.0 (7.2-16.9) | | 11.3 (7.4-16.1) | | | 0.105 |
| **Creatinine (mg/dL) (n=401)** | 1.1 (0.51-7.11) | | 1.05 (0.45-6.27) | | 1.3 (0.45-5.34) | | | 0.094 |

BMI: Body mass index; CK: creatinine kinase; COPD: chronic obstructive pulmonary disease; LVEF: left ventricular ejection fraction; MI: myocardial infarction; PCI: percutaneous coronary intervention. Values are shown in median (IQR) or %.
